# Identification and Characterization of an Oxidative Degradation Product of Fexofenadine, Development and Validation of a Stability-Indicating RP-UPLC Method for the Estimation of Process Related Impurities and Degradation Products of Fexofenadine in Pharmaceutical Formulations

**DOI:** 10.3797/scipharm.1111-07

**Published:** 2012-01-21

**Authors:** Bhupendrasinh Vaghela, Surendra Singh Rao, Annarapu Malleshwar Reddy, Panuganti Venkatesh, Navneet Kumar

**Affiliations:** 1 Dr. Reddy’s Laboratories Ltd., IPDO, Bachupally, Hyderabad-500072, A. P., India; 2 Department of Chemistry, J. J. T. University, Jhunjhunu, Rajasthan, India

**Keywords:** Fexofenadine, Validation, Stability-indicating, Forced degradation, Identification, characterization, UPLC

## Abstract

A novel stability-indicating gradient RP-UPLC method was developed for the quantitative determination of process related impurities and forced degradation products of fexofenadine HCl in pharmaceutical formulations. The method was developed by using Waters Aquity BEH C18 (100 mm x 2.1 mm) 1.7 μm column with mobile phase containing a gradient mixture of solvent A (0.05% triethyl amine, pH adjusted to 7.0 with ortho-phosphoric acid) and B (10:90 v/v mixture of water and acetonitrile). The flow rate of mobile phase was 0.4 mL/min with column temperature of 30°C and detection wavelength at 220nm. Fexofenadine HCl was subjected to the stress conditions including oxidative, acid, base, hydrolytic, thermal and photolytic degradation. Fexofenadine HCl was found to degrade significantly in oxidative stress conditions, and degradation product was identified and characterized by ESI-MS/MS, ^1^H and ^13^C NMR spectroscopic method as the N-oxide 2-[4-(1-hydroxy-4-{4-[hydroxy(diphenyl)methyl]-1-oxido-piperidin-1-yl}butyl)phenyl]-2-methylpropanoic acid. The degradation products were well resolved from fexofenadine and its impurities. The mass balance was found to be satisfactory in all the stress conditions, thus proving the stability-indicating capability of the method. The developed method was validated as per ICH guidelines with respect to specificity, linearity, limit of detection and quantification, accuracy, precision and robustness.

## Introduction

**Fig. 1.**
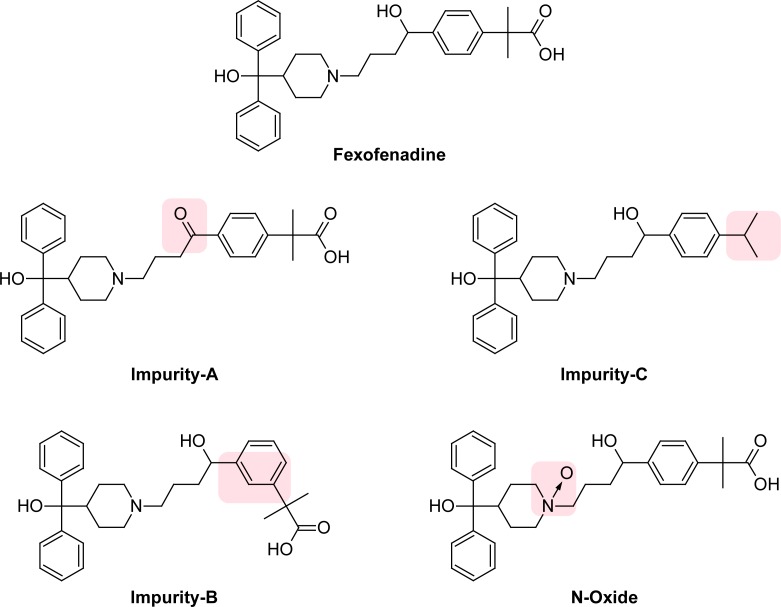
Structures and chemical names of Fexofenadine HCl and its impurities

Fexofenadine, 2-[4-(1-hydroxy-4-{4-[hydroxy(diphenyl)methyl]piperidin-1-yl}butyl)phenyl]-2-methylpropanoic acid (Fig. 1), is a highly selective peripheral histamine H1 receptor antagonist used in the treatment of allergic diseases such as allergic rhinitis and chronic urticaria. Fexofenadine is the active derivative of the antihistamine terfenadine, with no anti-chrolinergic or alpha 1-adernergic receptor-blocking effects and without severe cardiac side effects of terfenadine [1, 2].

According to the literature survey, few HPLC assay and dissolution methods have been reported for determination of fexofenadine in pharmaceutical preparation [3–6]. Estimation of fexofenadine in biological fluids using liquid chromatography with mass spectroscopy [7], ionspray tandem mass spectroscopy [8], electronspray tandem mass spectroscopy [9], UV detection [10, 11] and fluorescence detection [12] has been performed. Literature reported the isolation and structure elucidation of photodegradation products of fexofenadine [13] and photodegradation kinetics of fexofenadine HCl using LC method [14]. Methods are reported for the determination of fexofenadine and its two related compounds/degradation compound by HPLC [15, 16]. However, reported methods have not mentioned a new potential oxidative degradent which is N-oxide formation at tertiary amine of feofenadine and eluation of highly non polar related compound imp-c. In this method, we have observed one new potential impurity in our drug product during oxidative forced degradation study. Degradation product formed during oxidative stress was isolated and characterized by ESI-MS/MS,^1^H and ^13^C NMR as the N-oxide impurity, 2-[4-(1-hydroxy-4-{4-[hydroxy(diphenyl)methyl]-1-oxidopiperidin-1-yl}butyl)phenyl]-2-methyl-propanoic acid. To our present knowledge, this N-oxide impurity of fexofenadine is not reported in literature and there is no stability-indicating LC method available for the estimation of N-oxide impurity in pharmaceutical formulation. The present work describes the isolation and characterization of N-oxide impurity, as well as the development and validation of a stability-indicating RP-UPLC method for the estimation of degradation and process related impurities of fexofenadine hydrochloride, namely N-oxide, imp-A, imp-B and imp-C [Fig. 1]. The developed LC method was validated with respect to specificity, limit of detection and quantification, linearity, precision, accuracy and robustness. Forced degradation studies were performed on the placebo (all excipient mixture without fexofenadine HCl drug substance) and drug product to show the stability-indicating nature of the method. These studies were performed in accordance with established ICH guidelines [17, 18].

## Experimental

### Chemicals and reagents

Samples of fexofenadine hydrochloride tablets and its impurities were supplied by Dr. Reddy’s laboratories limited, Hyderabad, India. The HPLC grade acetonitrile, analytical grade triethyl amine and ortho-phosphoric acid were purchased from J.T.Baker, Mumbai, India. High purity water was prepared using Millipore Milli-Q Plus water purification system (Millipore, Milford, MA, USA).

### Equipment

Acquity UPLCTM (Water, Milforde, USA) was used which is equipped with a binary solvent manager, a sample manager and a photodiode array (PDA) detector. The output signals were monitored and processed using Empower 2 software. Cintex digital water bath was used for hydrolysis studies. Photo-stability studies were carried out in photo-stability chamber (Sanyo, Leicestershire, UK). Thermal stability studies were performed in a dry air oven (Cintex, Mumbai, India). The pH of the solutions was measured by a pH meter (Mettler-Toledo, Switzerland).

### Chromatographic Conditions

The method was developed using Waters Aquity BEH C18 (100 mm x 2.1 mm) 1.7 μm particle size column (Waters, Milforde, USA) with mobile phase containing a gradient mixture of solvent A (0.05% v/v triethyl amine, pH adjusted to 7.0 with ortho-phosphoric acid) and solvent B (mixture of water and acetonitrile in the ratio of 10:90 v/v, respectively). The gradient program (T/%B) was set as 0/25, 10/25, 15/35, 33/60, 35/80, 36/25 and 40/25. The flow rate of the mobile phase was set at 0.4 mL/min. The column temperature was maintained at 30° C and the eluted compounds were monitored at the wavelength of 220 nm. The sample injection volume was 1.5 μl.

### Semi-Preparative LC Conditions

The separation and isolation of the degradation products were carried out on a semi-preparative Inertsil ODS 3V (10 mm × 250 mm; particle size 5 μm) LC column using mobile phase containing solvent A (0.01 M ammonium formate buffer) and B (0.01 M ammonium formate buffer and acetonitrile in the ratio of 10:90 v/v) at a flow rate of 4.0 mL/min. The gradient program (Time (min)/%B) was set 0/20, 10/35, 25/50, 35/90, 40/100, 60/100, 65/20 and 75/20 and the detector was maintained at 220 nm.

### Liquid Chromatography-Mass Spectroscopy (LC-MS) Conditions

An LC-MS/MS system (Agilent 1100 series liquid chromatograph coupled with Applied Biosystem 4000 Q Trap triple quadrupole mass spectrophotometer with Analyst 1.4 software, MDS SCIEX, USA) was used for the confirmation of atomic mass number of unknown compounds formed during forced degradation studies. The method was developed using Zorbax SB Phenyl, 250 × 4.6 mm, 5μm column as stationary phase with mobile phase containing a gradient mixture of solvent A (0.01 M ammonium formate buffer) and B (0.01 M ammonium formate buffer and acetonitrile in the ratio of 10:90 v/v). Solvent B was used as diluent. The gradient program (Time (min)/%B) was set 0/30, 7/40, 20/50, 25/90, 27/100, 50/100, 52/30 and 60/30. Prior to use mobile phase was mixed thoroughly and degassed. The mobile phase pumped at a flow rate of 1.0 mL/min. The column temperature was maintained at 25°C. The injection volume for sample was 20μL. The analysis was performed in positive electro-spray/ positive ionization mode. The source voltage was 5000 V and source temperature was 450°C. GS1 and GS2 were optimized to 30 and 35 psi, respectively. The curtain gas flow was 20 psi.

### NMR Conditions

The ^1^H and ^13^C Nuclear magnetic resonance (NMR) spectra were recorded in DMSO-*d*_6_ at 500 MHz and 125MHz, respectively, using Varian Unity INOVA 500 MHz spectrometer (Bruker Biospin, Germany). The chemical shift values were reported on δ scale in ppm with respect to TMS (0.00ppm) and DMSO-d6 (δ 39.5 ppm) as internal standard, respectively.

### Preparation of system suitability Solution

Mixture of milli-Q water and acetonitrile in the ratio of 50:50 v/v containing 0.1% v/v ortho-phosphoric acid was used as diluent. A system suitability solution of imp-B and fexofenadine HCl with a concentration of 2.4 μg/mL and 1.2 mg/mL, respectively, was prepared by dissolving appropriate amount of drug in the diluent.

### Preparation of Standard Solution

Stock solution of fexofenadine HCl was prepared in diluent with a concentration of 960 μg/mL. Working standard solution was prepared from diluting above stock solution of fexofenadine HCl with final concentration of 2.4 μg/mL.

### Preparation of sample Solution

Tablet powder equivalent to 120 mg of fexofenadine HCl was dissolved in diluent with sonication for about 25 min to prepare a solution containing 1200 μg/mL drug. This solution was centrifuged at 10000 rpm for about 10 min.

### Method validation

The proposed method was validated by determining its performance characteristic regarding specificity, accuracy, precision, limit of detection and quantification, linearity, range and robustness [17, 18].

### System Suitability

System suitability was determined before sample analysis. Single injection of system suitability solution and duplicate injections of the standard solution containing 2.4 μg/mL fexofenadine HCl were injected. The acceptance criteria were USP tailing factor not more than 2.0, USP plate count not less than 5000, area similarity ratio between 0.9 to 1.1 for fexofenadine peak (from duplicate injections of standard preparation) and; resolution should be minimum 3.0 between imp-B and fexofenadine peaks (from system suitability solution).

### Specificity/stress studies

Specificity is the ability of the method to measure the analyte response in the presence of its potential impurities. The specificity of the developed LC method for fexofenadine HCl was carried out in the presence of its impurities and degradation products. Stress studies were performed at 1200μg/mL concentration of fexofenadine HCl on tablets to provide an indication of the stability-indicating property and specificity of proposed method. The stress condition employed for degradation study included acid hydrolysis (1 N HCl at 60°C for 3.5 hrs), base hydrolysis (2 N NaOH at 60°C for 24 hrs), oxidation (3% H_2_O_2_ at 60°C for 5 hrs), hydrolytic (water at 60°C for 24 hrs), thermal (105°C for 24 hrs), humidity (25°C/90% RH for 7 days) and photolytic degradation (drug product exposed to visible light for 240 h resulting in an overall illustration 1.2 million lux h and UV light for 250 h resulting an overall illustration 200 watt h/m^2^ at 25°C, [19]). Peak purity test was carried out for the fexofenadine peak by using PDA detector in stress samples.

Placebo interference was evaluated by analyzing the placebo prepared as per test method. No peak due to placebo detected at the retention time of fexofenadine and its impurities.

### Precision

The precision of method was verified by repeatability and intermediate precision. Repeatability was checked by injecting six individual preparations of fexofenadine HCl tablets spiked with its four impurities; N-oxide and imp-B at 0.10% level, imp-A at 0.2% level and imp-C at 0.15% level (% level of each impurity with respect to 1.2 mg/mL fexofenadine HCl). % RSD of area for each impurity was calculated. The intermediate precision of the method was also evaluated using different analyst and different instrument and performing the analysis on different days.

### Limit of Detection (LOD) and Quantification (LOQ)

The LOD and LOQ for fexofenadine HCl impurities were determined at a signal-to-noise ratio of 3:1 and 10:1, respectively, by injecting a series of dilute solutions with known concentrations. Precision study was also carried out at the LOQ level by injecting six individual preparations of fexofenadine HCl impurities and calculating the %RSD of the area.

### Linearity

Linearity test solutions were prepared by diluting the stock solutions to the required concentrations. The solutions of each impurity were prepared at six concentration levels from LOQ to 200% of specification level. Calibration curves were plotted between the responses of peak versus analyte concentrations. The coefficient correlation, slope and y-intercept of the calibration curve are reported.

### Accuracy

Accuracy of the method for N-oxide, imp-A, imp-B and imp-C was evaluated in triplicate using six concentration levels of LOQ, 50%, 75%, 100%, 125% and 150%. The percentage recoveries for each impurity were calculated.

### Robustness

To determine the robustness of the developed method, experimental conditions were deliberately altered and the resolution between imp-B and fexofenadine, and system suitability parameters for fexofenadine HCl standard were recorded. The variables evaluated in the study were pH of the mobile phase buffer (± 0.2), column temperature (± 5°C), flow rate (± 0.04 ml/min) and % organic in the mobile phase (± 10%).

### Solution stability and mobile phase stability

The solution stability of fexofenadine HCl and its impurities was determined by keeping test and standard solutions in tightly capped volumetric flasks at room temperature for up to 48 h and measuring the amount of four impurities at every 24 h interval against freshly prepared standard solution. The mobile phase stability was also determined by injecting freshly prepared solutions of fexofenadine HCl and its impurities at 24 h and 48 h. The mobile phase was not changed during the study.

## Results and Discussion

### Identification of N-oxide impurity

An unknown impurity with relative retention time (RRT) 0.71 with respect to fexofenadine was observed during oxidative degradation study. Fexofenadine (250 mg), dissolved in 20 mL of diluent and 10 mL 30% of hydrogen peroxide, was subjected to oxidative degradation at 80°C temperature for 8 h. About 70% fexofenadine was degraded and degradation product was isolated by semi-preparative HPLC. Fractions from the semi-preparative HPLC separations were collected and evaporated to dryness under vacuum at 40°C. The chromatographic purity of isolated degradation compound was found to be > 97% and used for its identification by LC-MS and NMR studies.

**Fig. 2.**
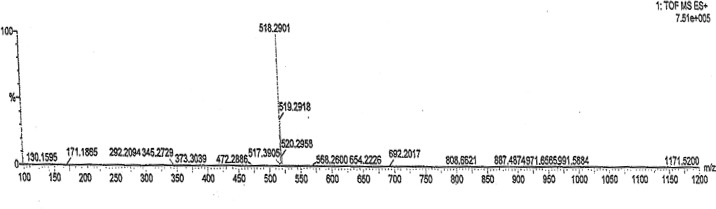
Mass spectrum of N-oxide impurity

ESI-MS mass spectral analysis (positive mode) (Fig. 2) of degraded compound showed a molecular ion at m/z 518 amu [M+H]^+^ which was 16 amu more than that of fexofenadine (m/z 502). This data indicated the presence of additional ‘O’ functionality in degradation compound. Use of high resolution mass spectroscopy (HRMS) in this measurement confirms elemental composition C_32_H_39_NO_5_ of the unknown degradant compound.

To get the structural information, degradation product was further subjected to ^1^H and ^13^C NMR study. The number of proton and carbon resonances is the same as that in Fexofenadine. However, the ^1^H and the ^13^C chemical shifts of the methylene groups attached to the nitrogen atom in the piperidine ring are deshielded when compared to those of Fexofenadine. Variations were observed in the ppm values (shifted slightly toward downfield) for the hydrogens present on piperidine ring and the aliphatic side chain attached to nitrogen atom. The ^13^C signals for the carbons of piperidine ring and the aliphatic side chain attached to nitrogen were also shifted slightly toward downfield as well (table 6). This observation lends support to the formation of N-Oxide in the piperidine ring. This is in agreement with the HRMS pattern observed for N-Oxide.

Identification of unknown compound was verified by ^1^H and ^13^C NMR study. The assignment of NMR signals were performed for unknown impurity and confirmed as 2-[4-(1-hydroxy-4-{4-[hydroxy(diphenyl)methyl]-1-oxidopiperidin-1-yl}butyl)phenyl]-2-methyl-propanoic acid.

### Method Development and Optimization of stability-indicating UPLC method

The main objective of the chromatographic method was to separate critical closely eluting compounds fexofenadine and imp-B and to elute non-polar imp-C with a shorter run time. The blend containing 1.2 mg/ml of fexofenadine and 2.4 μg/ml of each imp-A, imp-B, imp-C and N-oxide impurity was used for separation. A gradient elution method was employed using solvent A (0.05 M sodium dihydrogen orthophosphate and 0.01 M sodium perchlorate, pH 3.0) and acetonitrile as solvent B, Acquity BEH C8 (100 mm X 2.1 mm) 1.7μm column with flow rate of 0.5 mL/min on UPLC equipped with photo diode array detector. Resolution between fexofenadine and imp B was less than 2.0 and baseline disturbance was observed at the retention time of imp-C. To increase the resolution and baseline stabilization an attempt was made with modified solvent A (0.05% triethyl amine, pH adjusted to 7.0 with ortho-phosphoric acid) and solvent B (mixture of water and acetonitrile in the ratio of 10:90 v/v) and Acquity BEH C18 (100 mm X 2.1 mm) 1.7μm column. On the optimization of gradient program, fexofenadine and all four impurity peaks were well resolved from each other and degradation products. Based on these experiments, the final optimized conditions are described below.

Waters Aquity BEH C18 (100 mm × 2.1 mm) 1.7 μm particle size column was used as stationary phase. The mobile phase A consisted of 0.05% triethyl amine, pH adjusted to 7.0 with ortho-phosphoric acid and mobile phase B contained a mixture of water and acetonitrile in the ratio of 10:90 v/v, respectively. The gradient program (T/%B) was set as 0/25, 10/25, 15/35, 33/60, 35/80, 36/25 and 40/25. The flow rate of the mobile phase was set at 0.4 mL/min. The column temperature was maintained at 30° C and the eluted compounds were monitored at the wavelength of 220 nm. The sample injection volume was 1.5 μl.

## Method validation

The proposed method was validated as per ICH guidelines [17]. The following validation characteristics were addressed: specificity, accuracy, precision, linearity, range and robustness.

### System suitability

System suitability shall be checked for the conformance of suitability and reproducibility of chromatographic system for analysis. The system suitability was evaluated on the basis of USP plate counts, USP tailing factor, peak area ratio for fexofenadine peaks from standard solution and resolution from system suitability solution. All critical parameters tested met the acceptance criteria (Table 1).

### Specificity

**Fig. 3.**
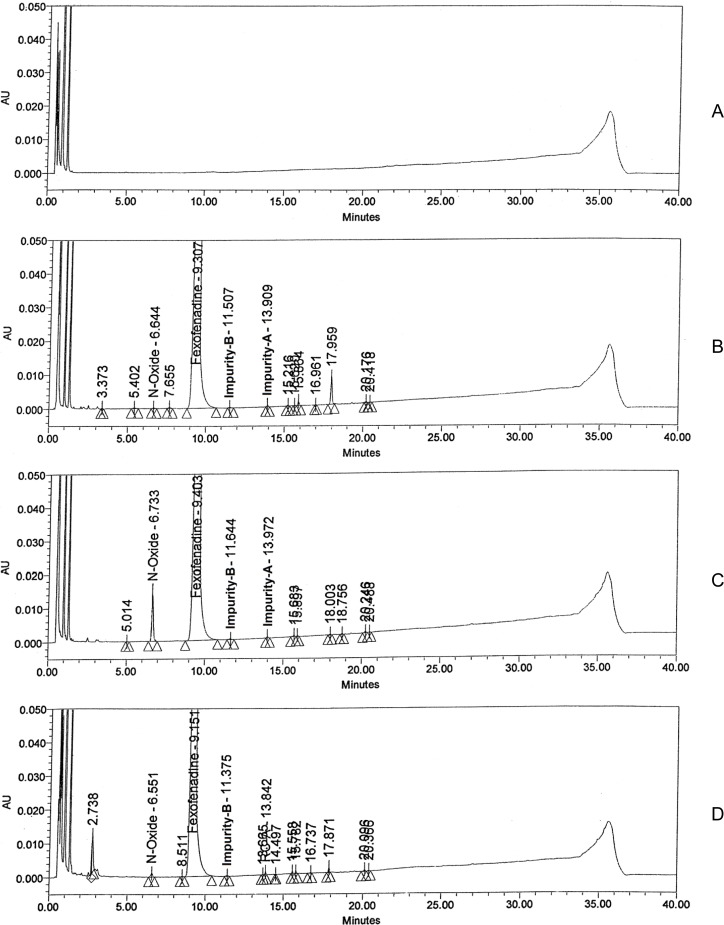

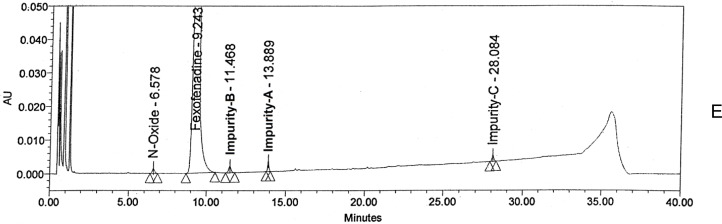
Typical chromatograms of (A) Placebo, (B) Acid degradation sample, (C) Peroxide degradation sample, (D) Thermal degradation sample and (E) Fexofenadine test spiked with its impurities

The aim of the specificity study is to assess unequivocally analyte in the presence of components that may be expected to be present. Placebo interference was evaluated by analyzing the placebo prepared as per test method. No peak due to placebo detected at the retention time of fexofenadine and its impurities (Fig. 3A). All force degradation samples were analyzed at 1200 μg/mL concentration of fexofenadine HCl using PDA detector to ensure the homogeneity and purity of fexofenadine peak. Significant degradation was observed in oxidation degradation (3% H_2_O_2_ at 60°C for 5 h) and slight degradation was observed in acid hydrolysis (1 N HCl at 60°C for 3.5 h), base hydrolysis (2 N NaOH at 60°C for 24 h) and thermal degradation (105°C for 24 h) (Fig. 3B–D). Oxidation degradation leads to the formation of a major unknown degradation product. Oxidative degradation product was identified and characterized by LC-MS/MS, ^1^H and ^13^C NMR as2-[4-(1-hydroxy-4-{4-[hydroxy(diphenyl)methyl]-1-oxidopiperidin-1-yl}butyl)phenyl]-2-methylpropanoic acid. Fexofenadine was found stable under hydrolytic (water at 80°C for 4 h), humidity (25°C/90% RH for 7 days) and photolytic (exposed to 1.2 million lux h visible light and 200 watt h/m^2^ UV light) degradation conditions. The mass balance (% assay + % sum of all degradants + % sum of all impurities) results were calculated and found to be more than 98.4% (Table 2). The purity of fexofenadine was unaffected by the presence of its impurities and degradation products and thus confirms the stability-indicating power of the developed method.

### Precision

The % RSD for the area of N-oxide, imp-A, imp-B and imp-C in repeatability study was within 3.0% and in intermediate precision study was within 3.6%, which confirms the precision of the method. The %RSD values are presented in Table 3.

### Limits of Detection and Quantification

The limit of detection, limit of quantification and precision at LOQ values for N-oxide, imp-A, imp-B and imp-C are reported in Table 3.

### Linearity

The linearity calibration plots for the N-oxide, imp-A, imp-B and imp-C was obtained over the calibration ranges tested, i.e. LOQ to 200% of specification level. The correlation coefficient obtained was greater than 0.998 and % bias at 100% response was within 5% (Table 3). The above result shows that a strong correlation exists between peak area and concentration of N-oxide, imp-A, imp-B and imp-C.

### Accuracy

The percentage recovery of N-oxide, imp-A, imp-B and imp-C in fexofenadine samples varied from 92.6 to 108.6%. The LC chromatogram of spiked sample at specification level of all four impurities in fexofenadine hydrochloride tablet sample is shown in Fig. 3E. The recovery values for fexofenadine impurities are presented in Table 4.

### Robustness

In all the deliberate varied chromatographic conditions (flow rate, column temperature, pH of mobile phase buffer and composition of organic solvent), all analytes were adequately resolved and elution order remained unchanged. The resolution between critical pair, i.e. for imp-B and fexofenadine was greater than 5.4 and tailing factor for fexofenadine peak from standard solution was not more than 1.0, and USP plate count was more than 11434 (Table 5).

### Stability of Solution and Mobile Phase

The variability in the estimation of all four fexofenadine impurities was within ± 10% during solution stability and mobile phase stability. The results from solution stability and mobile phase stability experiments confirmed that mobile phase was stable up to 48 h and sample solution and standard solutions were stable up to 48 h.

## Conclusion

This research paper describes the identification and characterization of a potential oxidative degradant (N-oxide) in fexofenadine hydrochloride in pharmaceutical formulations. The impurity was isolated by semi-preparative liquid chromatography. The isolated impurity was characterized by using spectroscopic techniques. A simple and efficient RP-UPLC method development and validation were discussed. The method was found to be precise, accurate, linear, robust and rugged during validation. The method is stability-indicating and can be used for routine analysis of production samples and to check the stability of the fexofenadine HCl tablets.

## Figures and Tables

**Fig. 4.**
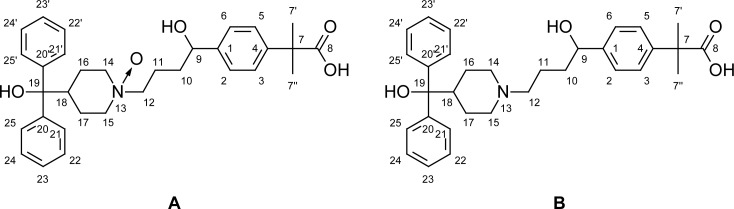
Structural formula and numbering of N-Oxide impurity (A) and Fexofenadine (B)

**Tab. 1.** System suitability test results

**Parameters**	**Specification**	**Observed values**	
		**Precision**	**Intermediate Precision**
Resolution^a^	≥ 3.0	7.3	6.9
Area ratio	≥ 0.9 and ≤ 1.1	1.0	1.0
USP Tailing	≤ 2.0	1.1	0.9
USP plate counts	≥ 5000	21047	14824

a Resolution between fexofenadine and Imp-B.

**Tab. 2.** Summary of forced degradation results

**Stress Condition**	**% Impurity**					**% Degradation**	**% Assay of active substance**	**Mass balance (%)**
	**N-oxide**	**Imp-A**	**Imp-B**	**Imp-C**	**MUSI**			
Acid hydrolysis	0.03	0.04	0.03	ND	0.49	0.60	97.8	98.4
Base hydrolysis	0.11	0.04	0.04	ND	0.03	0.18	100.5	100.7
Oxidation degradation	1.72	0.05	ND	ND	0.05	1.98	99.0	101.0
Thermal Degradation	0.12	0.06	0.03	ND	0.08	0.80	102.9	103.7
Water Degradation	ND	0.03	ND	ND	ND	0.03	101.6	101.6
Photolytic degradation	0.03	0.03	0.03	ND	0.03	0.00	101.1	101.1
Humidity Degradation	ND	0.02	ND	ND	ND	0.02	101.0	101.0

MUSI…Maximum un-specified impurity; ND…Not detected.

**Tab. 3.** Linearity and precision data

**Parameter**	**N-oxide**	**Imp-A**	**Imp-B**	**Imp-C**
LOD (μg/mL)	0.196	0.166	0.190	0.159
LOQ (μg/mL)	0.588	0.496	0.571	0.476
Correlation coefficient	0.999	0.999	0.998	0.999
Intercept (a)	361.70	13.66	249.36	−478.95
Slope (b)	5814.98	6080.89	5118.69	6830.94
Bias at 100% response	2	0	4	4
Precision (%RSD)	2.9	0.4	2.6	3.0
Intermediate precision (%RSD)	3.2	0.8	5.1	3.6
Precision at LOQ (%RSD)	3.3	2.4	4.8	2.8

**Tab. 4.** Recovery data

**Amount spiked^a^**	**% Recovery^b^**			
	**N-oxide**	**Imp-A**	**Imp-B**	**Imp-C**
LOQ	92.6 ± 2.3	104.9 ± 3.5	93.1 ± 3.4	94.1 ± 1.5
50%	107.0 ± 1.3	106.6 ± 0.6	107.8 ± 1.0	104.2 ± 3.5
75%	94.8 ± 1.9	108.6 ± 2.2	95.4 ± 2.8	97.2 ± 3.9
100%	96.7 ± 0.4	104.1 ± 0.6	95.1 ± 3.0	92.3 ± 2.6
125%	98.3 ± 1.2	102.3 ± 2.3	96.7 ± 1.7	94.3 ± 1.7
150%	103.0 ± 0.9	107.2 ± 1.0	107.5 ± 1.8	100.2 ± 0.9

a Amount of four impurities spiked with respect to specification level;

b Mean ± %RSD for three determinations.

**Tab. 5.** Robustness results of UPLC method

**Variation in chromatographic condition**	**Observed system suitability parameters**			
	**Area ratio ≥ 0.9 and ≤ 1.1**	**Resolution^a^ ≥ 3.0**	**USP Tailing ≤ 2.0**	**USP plate count ≥ 5000**
Column Temperature 25°C	1.0	5.9	0.9	14654
Column Temperature 35°C	1.0	6.3	0.9	16417
Flow rate 0.36 mL/min	1.0	6.0	1.0	16309
Flow rate 0.44 mL/min	1.0	6.0	1.0	11590
Acetonitrile 90%	1.0	5.5	0.9	23488
Acetonitrile 110%	1.0	5.9	0.9	11434
Mobile Phase Buffer pH 6.8	1.0	6.0	1.0	12061
Mobile Phase Buffer pH 7.2	1.0	5.4	1.0	13461

a Resolution between fexofenadine and Imp-B.

**Tab. 6.** NMR assignments of fexofenadine and N-oxide impurity.

**Position^a^**	**^1^H**	**N-Oxide Impurity**			**Fexofenadine**		

		**δ (ppm)**	**J(Hz)^b^**	**^13^C**	**δ (ppm)**	**J(Hz)^b^**	**^13^C**
1	–	–	–	144.2	–	–	144.1
2,6	2H	7.24–7.57	–	125.2	7.10–7.30	–	125.3
3,5	2H	7.51	d, 7.5	125.5	7.50	d, 7.4	125.7
4	–	–	–	143.8	–	–	143.5
7	–	–	–	45.7	–	–	45.5
7′,7″	6H	1.42	s	26.6	1.45	s	26.5
8	–	–	–	178.1	–	–	177.6
9	1H	4.51	t, 6.5	71.4	4.51	t, 6.5	71.3
10	2H	1.28	m	35.8	1.76	m	36.1
11	2H	1.56	m	20.8	1.76	m	20.0
12	2H	3.38	t,12.0	68.3	2.92	t, 12.0	56.1
14	2H	3.52	m	61.9	2.92	m	51.6
15	2H	3.52	m	62.0	3.44	m	51.6
16,17	Ha	1.80–1.92	m	22.5	1.76	m	24.0
	He	2.08	m	–		m	–
18	1H	2.83	t, 12.0	40.4	2.92	t, 12.0	40.9
19	–	–	–	78.1	–	–	78.3
20,20′	–	–	–	146.6	–	–	146.7
21,21′	2H	7.24–7.57	–	125.6	7.10–7.30	–	125.7
22,22′	2H	7.24–7.57	–	128.0	7.10–7.30	–	128.0
23,23′	2H	7.24–7.57	–	126.1	7.10–7.30	–	126.1
24,24′	2H	7.24–7.57	–	127.9	7.10–7.30	–	127.9
25,25′	2H	7.24–7.57	–	125.7	7.10–7.30	–	125.7

a Refer to Fig. 4 the structural formula for numbering;

b This column gives the ^1^H-^1^H multiplicity and coupling constants; s…Singlet; d…Doublet; t…Triplet; m…Multiplet.
